# Complete Genome Sequences of *Sweet potato feathery mottle virus* and *Sweet potato virus G* from Brazil

**DOI:** 10.1128/genomeA.01567-17

**Published:** 2018-02-01

**Authors:** Caroline A. Souza, Maurício Rossato, Fernando L. Melo, Rita C. Pereira-Carvalho

**Affiliations:** a Department of Phytopathology, Plant Virology Area, Universidade de Brasília, Brasília, Distrito Federal, Brazil; b Department of Cell Biology, Universidade de Brasília, Brasília, Distrito Federal, Brazil

## Abstract

In Brazil, *Potyvirus* species in sweet potatoes have been detected mostly by serology. Here, we report the complete genome sequences of two *Potyvirus* species, *Sweet potato feathery mottle virus* strain (SPFMV-UNB-01) and *Sweet potato virus G* strain (SPVG-UNB-01).

## GENOME ANNOUNCEMENT

The sweet potato (*Ipomoea batatas* Lam.) (1) is a tuberous root (2) belonging to the *Convolvulaceae* family. Globally, sweet potato is the seventh most important food crop after rice, wheat, potatoes, maize, barley, and cassava, but in developing countries, it is the fifth most important food crop (3).

The sweet potato is host to several pathogens, including synergistic viral complexes that trigger a more aggressive disease with considerable damages to the host (4, 5). There are currently 35 viral species that have been reported to infect sweet potato crops worldwide (6). In Brazil, 11 virus species have been reported to infect the sweet potato, of which 4 are of the genus *Potyvirus* (family *Potyviridae*), *Sweet potato feathery mottle virus* (SPFMV), *Sweet potato latent virus* (SPLV), *Sweet potato mild speckling virus* (SPMSV), and *Sweet potato virus G* (SPVG) (4).

In this study, 40 sweet potato plants from distinct producing regions in Brazil were grafted on *Ipomoea setosa* and kept in a greenhouse at the Experimental Station of Biology of the Universidade de Brasília (EEB-UnB). The virus enrichment process with semipurification of particles was carried out using leaves of *I. setosa*. Samples were weighed individually, to 1 g of each plant, and then pooled, forming a sample composed of 10 plants (10 g). From the purified particles, RNA extraction was performed using Trizol reagent following the manufacturer’s instructions. The composite sample was sent for sequencing on the Illumina MiSeq platform at the Universidade Católica de Brasília (UCB).

The raw reads (6,862,788 million paired-end reads) were trimmed and *de novo* assembled using CLC Genomics Workbench version 8.0. The resulting contigs were compared to the viral RefSeq database available in GenBank using a BLASTx search (7) implemented in Geneious version 9.1.5 (7). The contigs related to SPFMV (84 contigs) and SPVG (3 contigs) were selected and mapped against reference genomes. The resulting genome of SPFMV (10,810 nucleotides [nt] in length) encodes one polyprotein (3,493 amino acids [aa]) along with the PISPO (654 aa) and PIPO (218 aa) proteins, with 97.08% nt identity over the entire genome compared to the SPFMV reference (GenBank accession number NC_001841). The SPVG genome sequence was 10,775 nt in length with 98.31% nt identity compared to the SPVG reference genome (GenBank accession number NC_018093). The genome encodes a polyprotein (3,488 aa) and the PISPO (601 aa) and PIPO (216 aa) proteins. Phylogenetic analysis using complete genomes available in GenBank confirmed that both isolates are related to viruses from Asia and South America. These are the first complete genome sequences of SPFMV and SPVG from Brazil.

### Accession number(s).

The sequences reported here have been deposited in GenBank under the accession numbers MF185715 (strain SPFMV-UNB-01) and MF185716 (strain SPVG-UNB-01).
